# Porcine intestinal epithelial barrier disruption by the *Fusarium* mycotoxins deoxynivalenol and T-2 toxin promotes transepithelial passage of doxycycline and paromomycin

**DOI:** 10.1186/1746-6148-8-245

**Published:** 2012-12-17

**Authors:** Joline Goossens, Frank Pasmans, Elin Verbrugghe, Virginie Vandenbroucke, Siegrid De Baere, Evelyne Meyer, Freddy Haesebrouck, Patrick De Backer, Siska Croubels

**Affiliations:** 1 Department of Pharmacology, Toxicology and Biochemistry, Faculty of Veterinary Medicine, Ghent University, Salisburylaan 133, B-9820, Merelbeke, Belgium; 2 Department of Pathology, Bacteriology and Avian Diseases, Faculty of Veterinary Medicine, Ghent University, Salisburylaan 133, B-9820, Merelbeke, Belgium

**Keywords:** Mycotoxins, Cytotoxicity, Flow cytometry, Permeability, Intestinal cells

## Abstract

**Background:**

The gastrointestinal tract is the first target for the potentially harmful effects of mycotoxins after intake of mycotoxin contaminated food or feed. With deoxynivalenol (DON), T-2 toxin (T-2), fumonisin B1 (FB1) and zearalenone (ZEA) being important *Fusarium* toxins in the northern hemisphere, this study aimed to investigate *in vitro* the toxic effect of these mycotoxins on intestinal porcine epithelial cells derived from the jejunum (IPEC-J2 cells). Viability of IPEC-J2 cells as well as the proportion of apoptotic and necrotic IPEC-J2 cells was determined by flow cytometry after 72 h of exposure to the toxins. Correlatively, the integrity of the intestinal epithelial cell monolayer was studied using Transwell® inserts, in which the trans-epithelial electrical resistance (TEER) and passage of the antibiotics doxycycline and paromomycin were used as endpoints.

**Results:**

We demonstrated that the percentage of Annexin-V-FITC and PI negative (viable) cells, Annexin-V-FITC positive and PI negative (apoptotic) cells and Annexin-V-FITC and PI positive (necrotic) IPEC-J2 cells showed a mycotoxin concentration-dependent relationship with T-2 toxin being the most toxic. Moreover, the ratio between Annexin-V-FITC positive and PI negative cells and Annexin-V-FITC and PI positive cells varied depending on the type of toxin. More Annexin-V-FITC and PI positive cells could be found after treatment with T-2 toxin, while more Annexin-V-FITC positive and PI negative cells were found after exposure to DON. Consistent with the cytotoxicity results, both DON and T-2 decreased TEER and increased cellular permeability to doxycycline and paromomycin in a time- and concentration-dependent manner.

**Conclusions:**

It was concluded that *Fusarium* mycotoxins may severely disturb the intestinal epithelial barrier and promote passage of antibiotics.

## Background

Mycotoxins are naturally occurring secondary metabolites produced by fungi. They can be formed in the field on crops as well as during storage and are chemically very stable, enabling them to survive processing, and thereby end up in the feed and food chain. In the northern temperate regions *Fusarium* species are the most prevalent toxigenic fungi [1,2] with trichothecenes, fumonisins and zearalenone being the most important toxins produced [3]. If ingested, these mycotoxins can cause a variety of adverse health effects, both on humans and animals [4]. Since the gastrointestinal epithelium is the first barrier to come in contact with mycotoxins after ingestion, several studies investigated the effects of mycotoxins on intestinal cells. Most of these were conducted on human intestinal epithelial Caco-2 cells [5-16], but also the porcine intestinal epithelial cell lines IPEC-1 [17] and IPEC-J2 [18-20] have been used to demonstrate that mycotoxins can alter the integrity of the gut barrier. Moreover, Pinton *et al.*[12,13] showed that the trichothecene deoxynivalenol (DON) selectively affects the expression of tight junction proteins in porcine intestinal epithelial cell monolayers (IPEC-1) and Caco-2 cell monolayers resulting in increased paracellular passage of fluorescein isothiocyanate (FITC)-dextran and *Escherichia coli*. It was also demonstrated that fumonisin B_1 _(FB1) affects the membrane composition of IPEC-1 cells resulting in an increased passage of this toxin [21]. Intestinal epithelial cells are thus important targets for the toxic effects of mycotoxins and it is clear that an altered barrier could result in an altered passage of mycotoxin co-contaminants, xenobiotics and pathogens.

The aim of the present study was to evaluate the cytotoxic effect of the four most important *Fusarium* mycotoxins i.e. DON, T-2 toxin (T-2), zearalenone (ZEA) and FB1 in intestinal epithelial cells derived from the jejunum of pigs (IPEC-J2 cells). Therefore the percentage viable, apoptotic and necrotic IPEC-J2 cells was determined using flow cytometry. Correlatively, it was investigated whether differences in cell viability result in a changed transepithelial passage of the antibiotics doxycycline and paromomycin. This was studied using Transwell® devices which are commonly applied and widely accepted to perform permeability studies. The hypothesis tested in this study was that an altered integrity of the gut barrier provoked by mycotoxins, could result in an increased passage of antibiotics through the epithelium. Increased passage of these antibiotics can indeed have toxic consequences for the animal, withdrawal time, and consequently public health with respect to possible residues of drugs in edible tissues. To our knowledge, this is the first time that transport of antibiotics is used as a marker to study possible damage of mycotoxins to intestinal epithelial cells.

## Methods

### Cell line and culture conditions

The IPEC-J2 cell line is a continuous intestinal cell line originally derived from jejunal epithelia isolated from a neonatal, unsuckled piglet and maintained as a continuous culture [22]. Cells were grown in Dulbecco’s Modified Eagle Medium (DMEM)/Ham’s F12 (1:1) medium (Invitrogen™ Life Technologies, Carlsbad, CA, USA), supplemented with 5% fetal calf serum (FCS, HyClone, Cramlington, England, UK), 1% (v/v) insulin/transferrin/Na-selenite (Gibco, Life Technologies, Paisley, Scotland), 1% (v/v) penicillin/streptomycin (Gibco, Life Technologies) and 1% (v/v) kanamycin (Gibco, Life Technologies), further referred to as culture medium. The cells were routinely seeded at a density of 1.5 × 10^5 ^cells/ml with 18 ml medium in plastic tissue culture flasks (75 cm^2^, Nunc, Denmark), maintained in a humidified incubator at 37°C under 5% CO_2_, and passaged every 2–3 days. All experiments were performed with cells within maximal 10 passages.

### Chemicals

DON, T-2, FB1 and ZEA (Sigma-Aldrich, Bornem, Belgium) stock solutions of 2000 μg/ml were prepared in methanol for DON, ethanol for T-2 and acetonitrile for FB1 and ZEA (Merck, Darmstadt, Germany). Doxycycline and paromomycin stock solutions (Sigma-Aldrich, 2000 μg/ml) were prepared in high-performance liquid chromatography (HPLC) grade water. All stock solutions were stored at −20°C. Serial dilutions were prepared in culture medium in order to obtain non-cytotoxic concentrations of organic solvents and antibiotics, and allowing the addition of a similar volume of vehicle in all experiments.

### Determination of non-cytotoxic concentrations of the organic solvents

IPEC-J2 cells were seeded into a 96-well plate at a concentration of 1.5 × 10^5 ^cells/ml in a total volume of 200 μl. The cells were maintained in an atmosphere of 5% CO_2 _at 37°C. After overnight incubation, the cells were treated for 72 h with a final concentration of 1, 5, 10 or 20% of the organic solvents (methanol, ethanol or acetonitrile) in the total volume of culture medium. Next, to assess cytotoxicity, 10 μl of a water soluble tetrazolium salt (2-(4-iodophenyl)-3-(4-nitrophenyl)-5-(2,4-disulfophenyl)-2H-tetrazolium) (WST-1) was added to each well and the plate was incubated at 37°C for an additional 3 h. The absorbance was determined at 450 nm using a microplate ELISA reader (Multiscan MS, Thermo Labsystems, Helsinki, Finland). Each experiment was conducted in triplicate. The percentage of viable cells was calculated using the following formula:

$$\%\;\text{viability}=100\times \left(\left(c-b\right)–\left(a-b\right)\right)/\left(c-b\right)$$

In this formula a = OD_450nm _derived from the wells incubated with solvent, b = OD_450nm _derived from blank wells, c = OD_450nm _derived from untreated control wells.

### Assessment of cytotoxicity after exposure of IPEC-J2 cells to the investigated antibiotics doxycycline and paromomycin

A flow cytometric technique was used to assess the direct cytotoxic effect of the antibiotics doxycycline and paromomycin. IPEC-J2 cells were seeded at a concentration of 5 × 10^5 ^cells/ml in 24-well plates in 1 ml of culture medium and allowed to grow for 24 h. Next, cells were treated for 24 h with 0, 5, 10, 20 and 40 μg/ml of doxycycline or paromomycin. Each experiment was conducted in triplicate. After the incubation period, cells were trypsinized, culture medium and cells were collected, centrifuged for 5 min at 524 × *g* and 4°C and resuspended in 0.5 ml Hank’s Buffered Salt Solution (HBSS, Gibco). This procedure was repeated three times in order to remove cellular debris and antibiotics. Cell death was assessed using dual staining with Annexin-V-fluorescein isothiocyanate (Annexin-V-FITC, Roche Diagnostics, Belgium) and propidium iodide (PI, Sigma-Aldrich) which allows the discrimination of viable cells (FITC^-^/PI^-^), apoptotic (FITC^+^/PI^-^) and necrotic cells (FITC^+^/PI^+^). Cells were incubated for 20 min in the dark on ice with 100 μl of a solution containing 20 μl Annexin-V-FITC and 20 μl PI dissolved in 1000 μl incubation buffer containing 10 mM 4-(2-hydroxyethyl)-1-piperazineethanesulfonic acid (HEPES), 140 mM NaCl and 5 mM CaCl_2_. Cells were assessed by a FACSCanto flow cytometer (Becton, Dickinson and Company, Erembodegem, Belgium) and the percentage of viable, apoptotic and necrotic cells was obtained using the FACSDiva Software (Becton, Dickinson and Company, Franklin Lakes, NJ, USA).

### Assessment of cytotoxicity after exposure of IPEC-J2 cells to *Fusarium* toxins

The flow cytometric technique as described above was also used to assess the direct cytotoxic effect of mycotoxins. IPEC-J2 cells were seeded at a concentration of 5 × 10^5 ^cells/ml in 24-well plates in 1 ml of culture medium and allowed to grow for 24 h. Next, cells were treated for 72 h with increasing concentrations of T-2 (0–10 ng/ml), DON (0–10 μg/ml), ZEA (0–20 μg/ml) and FB1 (0–15 μg/ml). The molar concentration corresponding with 1 μg/ml DON, FB1 and ZEA and 1 ng/ml T-2 is 3.37 μM, 1.39 μM, 3.14 μM and 2.14 nM, respectively. Each experiment was conducted in triplicate. After the incubation period, cells were processed as described above before analysis on the FACSCanto flow cytometer. The concentration corresponding to 50% reduction in viable cells (IC50) was calculated with linear regression curves. A positive control was provided by cells single stained with Annexin-V-FITC and cells single stained with PI, also used to determine spectral overlap, after treatment for 72 h with 10 μg/ml DON. Furthermore, unstained cells (no Annexin-V-FITC and no PI) were also included to determine autofluorescence of IPEC-J2 cells.

### Assessment of TEER after exposure of IPEC-J2 cells to DON and T-2

The effect of DON and T-2 on barrier integrity was determined by measuring the transepithelial electrical resistance (TEER) using a Millicell Electrical Resistance System (Millipore, World Precision Instruments, Sarasota, FL, USA). Cells were grown and differentiated on Transwell® collagen-coated polytetrafluoroethylene (PFTE) membrane inserts (0.4 μm pore diameter, 6.5 mm diameter, Corning Inc., NY, USA) at a density of 1.5 × 10^5 ^cells/ml. Culture medium was replaced three times a week and IPEC-J2 cells were used for TEER experiments at day 21 post-seeding. Next, cells were incubated for 72 h with different concentrations of T-2 (0–100 ng/ml) or DON (0–10 μg/ml). TEER was measured every 24 h from the start of the toxin exposure until 3 days. TEER values were expressed as kΩ × cm^2^. Each single experiment was conducted in threefold.

### Passage of doxycycline and paromomycin after exposure of IPEC-J2 cells to DON and T-2

Cells were seeded on Transwell® collagen-coated PFTE membrane inserts as described above for the TEER experiment. Only cells which are cultured for 21 days show stable TEER values indicating cells have reached confluence and form a tight monolayer (data not shown). Next, cells were incubated for 72 h with different concentrations of T-2 (0–100 ng/ml) or DON (0–10 μg/ml), during which the last 24 h a non-cytotoxic concentration of both paromomycin (30 μg/ml) and doxycycline (10 μg/ml) was added to the apical (AP) compartment of the transwell. To evaluate the permeability of the monolayers, 50 μl of transport medium was withdrawn from the basolateral (BL) compartment at time 0 (before) and at 0.5, 1, 2, 3, 4, 6, 8, 12 and 24 h after addition of the drugs and replaced immediately by 50 μl of fresh culture medium. Each experiment was conducted in triplicate.

Acceptor media were analyzed for the paromomycin content by high-performance liquid chromatography tandem mass spectrometry (LC-MS/MS). The LC-MS/MS system consisted of a Surveyor Autosampler Plus and MS Pump Plus, coupled to a TSQ Quantum Ultra® triple quadrupole mass spectrometer (all from ThermoFisher Scientific, Zellik, Belgium). Chromatographic separation was performed on a PLRP-S column (150 mm x 2.1 mm i.d., 100 Å, dp: 5 μm, Varian, Middelburg, The Netherlands) using a gradient programme (flow-rate: 200 μl/min). The mobile phase consisted of (A) 20 mM pentafluoropropionic acid (PFPA) (Sigma-Aldrich) in water and (B) 20 mM PFPA in water/acetonitrile (50/50, v/v) (VWR International, Leuven, Belgium). The MS instrument was operated in the selected reaction monitoring (SRM) mode, using two precursor ion > product ion transitions. Tobramycin (Sigma-Aldrich) was used as internal standard (IS). Samples were prepared by pipetting 50 μl culture medium into an Eppendorf cup (Novolab, Geraardsbergen, Belgium). Each sample was spiked with 10 μl of the IS working solution of 1 μg/ml in HPLC grade water (VWR International) and diluted with 65 μl methanol, 100 μl HPLC grade water and 25 μl 200 mM PFPA. After vortexing, samples were centrifuged at 7 800 × *g* for 10 min to remove possible solid particles. The sample was transferred into screw-capped conical vials and 5 μl were injected onto the LC-MS/MS system.

Doxycycline concentrations were determined using the same LC-MS/MS configuration and HPLC column as for paromomycin analysis. The mobile phase consisted of (A) 0.5% acetic acid in water and (B) acetonitrile. A gradient programme was applied and the flow-rate was 200 μl/min. The MS instrument was operated in the SRM mode, using two precursor ion > product ion transitions. Demethylchlortetracycline (DMCTC, Sigma-Aldrich) was used as IS. Samples were prepared by pipetting 25 μl culture medium into an Eppendorf cup. Each sample was spiked with 12.5 μl of the IS working solution of 10 μg/ml in HPLC grade water, followed by the addition of 75 μl 0.5% formic acid in HPLC grade water. After vortexing briefly and centrifugation, 5 μl were injected onto the LC-MS/MS system.

The limit of quantification (LOQ) was 10 ng/ml for both doxycycline and paromomycin. A detailed description of both methods can be found in Goossens *et al. *[23].

### Statistical analysis

Data were analyzed using one-way analysis of variance (ANOVA) to address the significance of difference between mean values with significance set at P < 0.05.

## Results

### A concentration of 1% ethanol, 5% methanol or 5% acetonitrile of the total volume of culture medium was determined to be non-cytotoxic for IPEC-J2 cells

**Figure 1:**
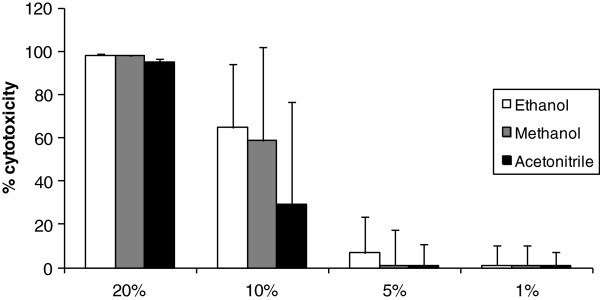
Percentage of cytotoxicity after exposure of IPEC-J2 cells for 72 h to 1, 5, 10 or 20% ethanol, methanol or acetonitrile in the total volume of culture medium (n=3, mean + stdev).

Figure 1 represents the percentage of viable cells after exposure for 72 h to 1, 5, 10 or 20% ethanol, methanol or acetonitrile in the total volume of culture medium.

A final concentration of 5% acetonitrile, 5% methanol and 1% ethanol was accepted to use in the experiment as the viability of IPEC-J2 cells was on average > 95%. Non-cytotoxic concentrations of the solvents were ensured by appropriate dilution of the toxins stock solutions in culture medium.

### A concentration of 10 μg/ml doxycycline and 30 μg/ml paromomycin was determined to be non-cytotoxic for IPEC-J2 cells

**Figure 2:**
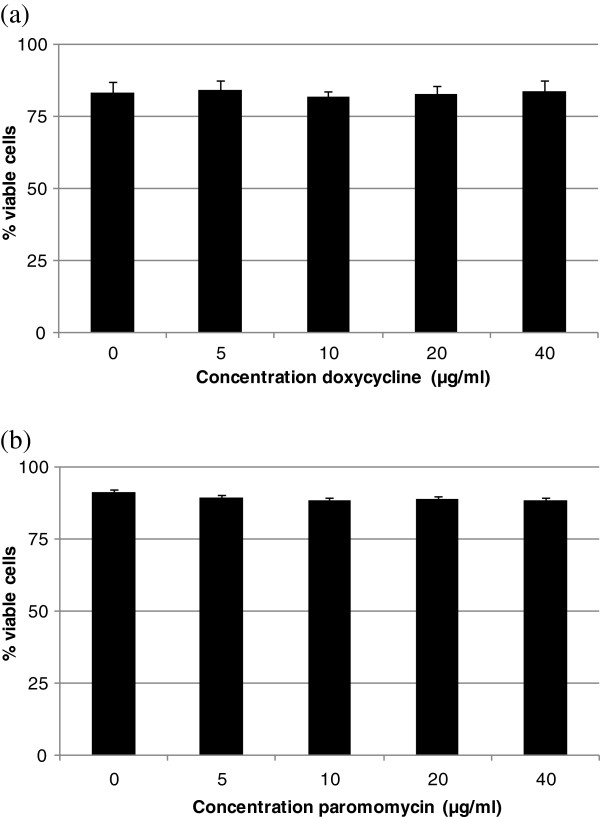
Percentage of viable cells after exposure of IPEC-J2 cells for 24 h to 0, 5, 10, 20 or 40 μg/ml doxycycline (a) or paromomycin (b) (n=3, mean + stdev).

Figure 2 represents the percentage of viable cells after exposure for 24 h to 5, 10, 20 or 40 μg/ml doxycycline (a) or paromomycin (b), respectively.

A concentration up to 40 μg/ml doxycycline or paromomycin caused no significant reduction in cell viability of IPEC-J2 cells. It was concluded that a concentration of 10 μg/ml doxycycline and 30 μg/ml paromomycin could be used in the experiments. The concentration of the antibiotics was based on, on the one hand, the dose of the antibiotic that is usually administered to pigs, and on the other hand, the capacity of the intestine of an adult pig. When 10 mg/kg body weight doxycycline and 40 mg/kg body weight paromomycin is administered to a pig of 20 kg, and this dose ends up in an intestinal volume of 27.5 l, this corresponds with a concentration of about 8 and 29 μg/ml respectively.

### Induction of necrosis and apoptosis in IPEC-J2 cells is *Fusarium* toxin dependent

**Figure 3:**
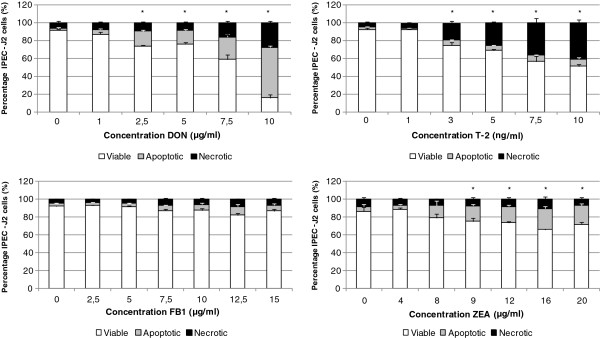
**Percentage viable, apoptotic and necrotic cells after incubation of undifferentiated IPEC-J2 cells for 72 h with different concentrations of DON, T-2, FB1 or ZEA (n=3, mean + stdev).** *Significant difference in viable cells compared to control (P < 0.05). The molar concentration corresponding with 1 μg/ml DON, FB1 and ZEA and 1 ng/ml T-2 is 3.37 μM, 1.39 μM, 3.14 μM and 2.14 nM, respectively.

Figure 3 represents the percentages of Annexin-V-FITC and PI negative, Annexin-V-FITC positive and PI negative and Annexin-V-FITC and PI positive IPEC-J2 cells, exposed for 72 h to different concentrations of DON, T-2, FB1 or ZEA, respectively.

Exposure of IPEC-J2 cells for 72 h to different mycotoxin concentrations showed a concentration-dependent toxicity. T-2 was clearly the most toxic one with a significantly lower percentage of Annexin-V-FITC and PI negative cells in the ng/ml range compared to DON and ZEA which were toxic in the μg/ml range.

FB1 was not toxic at the tested concentrations. For all mycotoxins, except T-2 toxin, a higher percentage of Annexin-V-FITC positive and PI negative cells rather than Annexin-V-FITC and PI positive cells can be found after exposure of undifferentiated IPEC-J2 cells for 72 h to different toxin concentrations. For T-2 toxin, the majority of dead cells were Annexin-V-FITC and PI positive. The IC50 for DON and T-2 was determined to be 6.98 μg/ml and 9.55 ng/ml, respectively.

### Cytotoxic concentrations of DON and T-2 induce a decrease in transepithelial electrical resistance (TEER) across the IPEC-J2 monolayer

**Figure 4:**
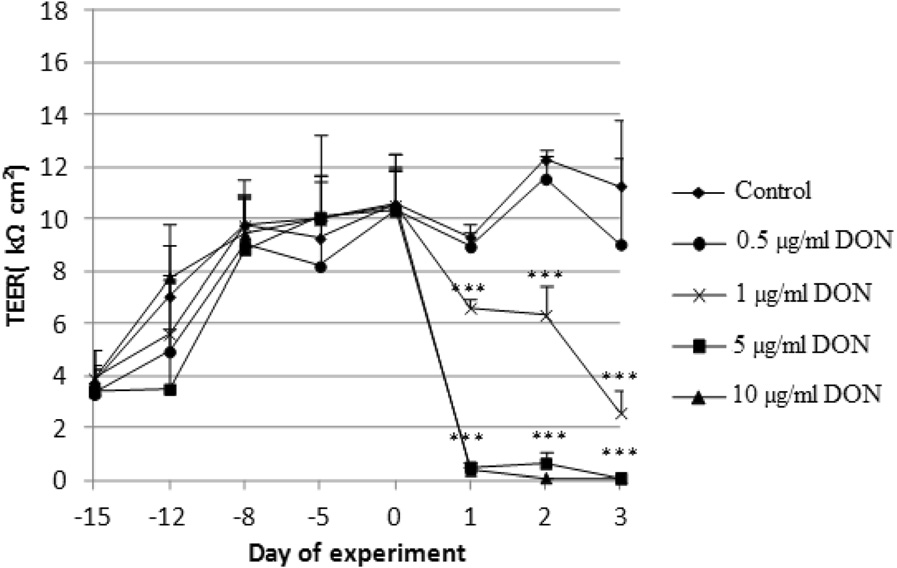
**Effect of DON on trans-epithelial electrical resistance (TEER) of differentiated IPEC-J2 cells.** Cells were grown for 21 days on Transwell® collagen-coated PTFE filters (0.33 cm^2^, 0.4 μm). Next, at day 0 various concentrations of DON were added to the apical compartment for 72 h: untreated inserts, inserts treated with 0.5, 1, 5 or 10 μg/ml of DON. TEER values are expressed as mean + stdev (n = 3) . Significant differences in TEER value have been marked with *** (P < 0.001).

**Figure 5:**
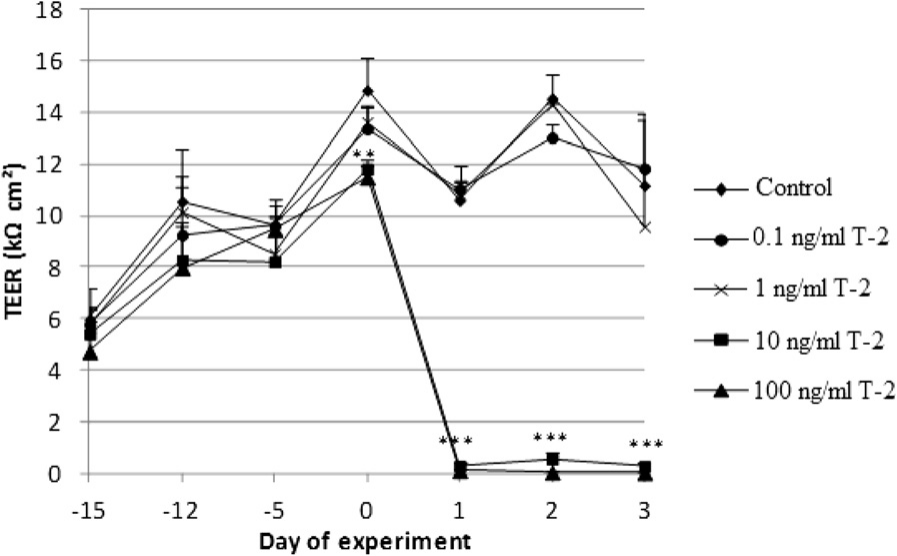
**Effect of T-2 on trans-epithelial electrical resistance (TEER) of differentiated IPEC-J2 cells.** Cells were grown for 21 days on Transwell® collagen-coated PTFE filters (0.33 cm^2^, 0.4 μm). Next, at day 0 various concentrations of T-2 were added to the apical compartment for 72 h: untreated inserts, inserts treated with 0.1, 1, 10 or 100 ng/ml of T-2. TEER values are expressed as mean + stdev (n = 3)**.** Significant differences in TEER value have been marked with ** (P < 0.01) or *** (P < 0.001) according to the significance level.

Figures 4 and 5 represent the effect of DON and T-2 on TEER of differentiated IPEC-J2 cells, respectively.

Both for T-2 and DON, a drop in TEER can be seen 24 h after incubation of IPEC-J2 cells with cytotoxic concentrations of the mycotoxins. Treatment with non-cytotoxic concentrations results in TEER values comparable to those of the control cells. Treatment of differentiated IPEC-J2 cells with 1 μg/ml DON shows a time-dependent decline in TEER.

### Disruption of epithelial integrity of the IPEC-J2 cell monolayer results in increased passage of doxycycline and paromomycin

**Figure 6:**
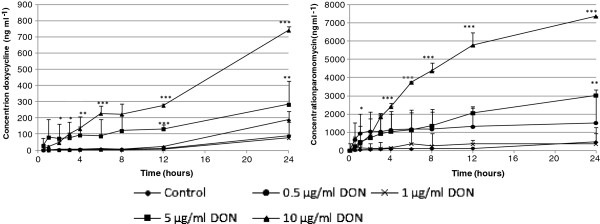
**Passage of doxycycline (a) and paromomycin (b) across a differentiated IPEC-J2 cell monolayer, measured as the concentration in the basolateral compartment.** Cells were grown for 21 days on Transwell® collagen-coated PTFE filters (0.33 cm^2^, 0.4 μm). Next, at day 0, cells were incubated for 72 h with different concentrations of DON (untreated inserts, inserts treated with 0.5, 1, 5 or 10 μg/ml), during which the last 24 h a non-cytotoxic concentration of doxycycline and paromomycin was added to the apical compartment (n = 3, mean + stdev). Significant differences in passage have been marked with * (P < 0.05), ** (P < 0.01) or *** (P < 0.001) according to the significance level.

**Figure 7:**
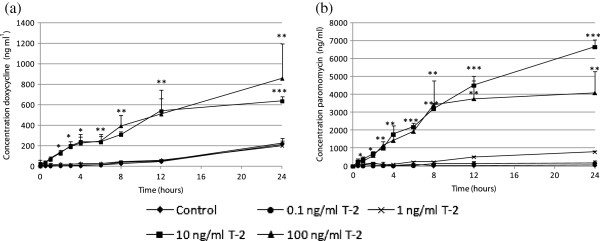
**Passage of doxycycline (a) and paromomycin (b) across a differentiated IPEC-J2 cell monolayer, measured as the concentration in the basolateral compartment.** Cells were grown for 21 days on Transwell® collagen-coated PTFE filters (0.33 cm^2^, 0.4 μm). Next, at day 0, cells were incubated for 72 h with different concentrations of T-2 (untreated inserts, inserts treated with 0.1, 1, 10 or 100 ng/ml of T-2), during which the last 24 h a non-cytotoxic concentration of doxycycline and paromomycin was added to the apical compartment (n = 3, mean + stdev). Significant differences in passage have been marked with * (P < 0.05), ** (P < 0.01) or *** (P < 0.001) according to the significance level.

Figures 6 and 7 represent the passage of doxycycline and paromomycin across the IPEC-J2 cell monolayer treated for 72 h with several concentrations of DON and T-2, respectively.

In both the T-2 and DON experiment, increased passage of doxycycline and paromomycin was seen after incubation of IPEC-J2 cells with cytotoxic concentrations of the tested mycotoxins.

## Discussion

The gastrointestinal tract can be exposed to high concentrations of mycotoxins after ingestion of mycotoxin contaminated food or feed. Since swine are the most sensitive domestic animal species to mycotoxins [24], this study aimed to investigate the toxic effect of four common *Fusarium* toxins, T-2, DON, ZEA and FB1 on IPEC-J2 cells. Studies concerning the effects of mycotoxins on IPEC-J2 cells are limited. The concentrations of the mycotoxins tested in this study are the result of several optimization experiments performed to refine the concentration range to be used for these *in vitro* experiments. According to Sergent *et al.*[14] DON concentrations in human intestine are estimated between 0.16 μg/ml and 2 μg/ml depending on the contamination level of the feed, including thus our tested range. Mycotoxin concentrations in the test solutions are not based on levels known to cause problems in pigs, but are based on the cytotoxicity results of the flow cytometer experiments, the solubility of the mycotoxin in aqueous solvents, and cost of the mycotoxin.

*In vitro* cell viability assays have a central role in predictive toxicology. The capability of commonly used colorimetric assays, such as the WST-1, MTT (3-(4,5-dimethyltiazol-2-yl)-2,5-diphenyltetrazolium bromide) and MTS assay (3-(4,5-dimethylthiazol-2-yl)-5-(3-carboxymethoxyphenyl)-2-(4-sulfophenyl)-2H-tetrazolium) or the lactate dehydrogenase (LDH) bioassay to detect cytotoxic effects is, however, limited. Cells that are apoptotic remain undetected with these assays. Our study used a flow cytometric technique, which allows to distinguish Annexin-V-FITC and PI negative (viable) cells from Annexin-V-FITC positive and PI negative (apoptotic) cells and Annexin-V-FITC and PI positive (necrotic) cells.

After 72 h of exposure with T-2, DON, FB1 or ZEA, T-2 clearly was the most cytotoxic with a toxicity in the ng/ml range, while the other mycotoxins are only toxic at concentrations in the μg/ml range. This finding is in accordance with the study of Calvert *et al. *[7], who demonstrated that T-2 was most cytotoxic against three human cell lines tested as determined by the MTT assay.

When comparing the percentage Annexin-V-FITC positive and PI negative cells and Annexin-V-FITC and PI positive cells, it is striking that more Annexin-V-FITC and PI positive cells than Annexin-V-FITC positive and PI negative cells were found after treatment of the cells with T-2. This corresponds, however, with findings in practice where T-2, present in feed, causes skin necrosis [25,26]. Also necrosis of the lymphoid cells of the intestinal mucosa was seen after intake of T-2 contaminated feed [25,27]. DON, on the other hand, causes more Annexin-V-FITC positive and PI negative cells than Annexin-V-FITC and PI positive cells. The discrepancy between type A (T-2) and type B (DON) trichothecenes was also demonstrated by Nasri *et al. *[28] who showed that DON causes rather apoptosis and T-2 rather necrosis in Jurkat T-lymphocytes. As it is known that type B trichothecenes activate the MAPK signaling pathway resulting in apoptosis, while type A trichothecenes are not able to activate the MAPK families [29-31], the authors suggested this as an explanation for the results. For ZEA and FB1, although FB1 was not cytotoxic at the tested concentrations, an approximately equal percentage of apoptotic and necrotic cells were seen after treatment of IPEC-J2 cells for 72 h with those toxins. Our results suggest that different toxins may induce different types of cell death in IPEC-J2 cells. To our knowledge, this is the first report of DON and T-2 induced different mechanisms of cell death in IPEC-J2 cells. Since it was not the principal aim of this study to elaborate further the differences in the mode of cell death induced by different mycotoxins, no further experiments were conducted. Nevertheless, it could be interesting to investigate these differences in more detail, including the investigation of associated pathway molecules, the analysis of potential protective effects of caspase inhibitors and/or necrose inhibitors, etc. Also other types of cell death can be taken into account since there are more than two ways for a cell to die. Cell death can be classified according to its morphological appearance (apoptotic, necrotic, autophagic or associated with mitosis), enzymological criteria (with and without the involvement of nucleases or distinct classes of proteases, such as caspases, calpains, cathepsins and transglutaminases), functional aspects (programmed or accidental, physiological or pathological) or immunological characteristics (immunogenic or non-immunogenic) [32]. It could be interesting exploring these differences in relation with mycotoxins.

Correlatively with direct cytotoxicity, the consequences on barrier integrity (TEER and passage of drugs) were also assessed. The effect on TEER and passage was only studied for DON and T-2 because, of the four mycotoxins tested in this study, these are the most toxic ones and their ratio of Annexin-V-FITC positive and PI negative cells and Annexin-V-FITC and PI positive cells is antithetic. Notwithstanding the assertion of Geens and Niewold [33] that IPEC-J2 cells cultured on Transwell®-collagen coated inserts with a surface area of 0.33 cm^2 ^are not appropriate to measure TEER values, our IPEC-J2 monolayer showed TEER values above 10 kΩ cm^2 ^after 3 weeks of culturing. According to Fromter and Diamond [34], IPEC-J2 cell lines with TEER values of approximately 2 kΩ cm^2 ^can be considered to be tight epithelia. When IPEC-J2 cells were incubated with cytotoxic concentrations of DON (5 and 10 μg/ml) and T-2 (10 and 100 ng/ml) TEER values dropped to nearly 0 kΩ cm^2 ^after 24 h suggesting a complete disruption of the epithelial monolayer within 24 h. In contrast, TEER values of IPEC-J2 cells incubated with non-toxic concentrations (0.5 μg/ml DON and 0.1 and 1 ng/ml T-2) were comparable to the TEER of control cells. Treatment with 1 μg/ml DON on the other hand shows a time-dependent reduction of the TEER. This was also reported by Pinton *et al. *[12] who showed a time- and dose-dependent reduction of the TEER after exposure of IPEC-1 and Caco-2 cells to different concentrations (0, 3, 6, 15, 30 μg/ml) of DON. A decreased barrier function *in vitro* is reflected in both a decreased TEER and an increased tracer flux [17,35]. In our study, the decrease in TEER is related to the passage of the used tracers, doxycycline (molecular weight (mw) 513 Da) and paromomycin (mw 714 Da), two commonly used antibiotics in pig industry. The higher the toxin concentration, the higher the marker passage. Most studies use tracers such as FITC-dextran, while in this study and for the first time the antibiotics doxycycline and paromomycin were used as markers. We were, however, not capable of demonstrating that induction of more apoptosis or more necrosis results in differences in transepithelial passage of antibiotics. Both doxycycline and paromomycin pass the epithelial monolayer, regardless the induction of a different cell death mechanism.

It is very interesting to study the passage of these therapeutic compounds during *in vivo* trials, as veterinary drugs are often used in pig mass medication and frequently administered by the oral route, i.e. mixed in the feed or drinking water.

## Conclusions

When discussing the relevance of the *in vitro* results for the *in vivo* situation it needs to be taken into account that enterocytes are not continuously exposed to mycotoxins. Rather a time-dependent fluctuation in mycotoxin concentrations exists *in vivo*, whereas *in vitro* cells are exposed continuously at constant concentrations. Nevertheless, the current *in vitro* findings warrant further investigation *in vivo* because an increased passage of antibiotics may have important implications for both animal and human health. Increased absorption of antibiotics may lead to higher plasma levels, including the risk of exceeding the toxic threshold. Moreover, increased antibiotic plasma levels may also lead to a tissue residue problem in slaughter animals and inherent food safety concerns. It could thus be interesting to investigate *in vivo* the effect of mycotoxins on the intestinal absorption of antibiotics.

## Competing interest

The authors declare that they have no competing interest.

## Authors’ contributions

JG drafted the manuscript and performed all experiments; VV and EV contributed to the manuscript; SDB helped with the determination of doxycycline and paromomycin plasma concentrations and helped to draft the manuscript; FP and EM participated in study coordination and helped to draft the manuscript. FH and PDB helped to draft the manuscript. SC supervised the project and helped to draft the manuscript. All authors have read and approved the final manuscript.
